# A Paraurethral Aggressive (Deep) Angiomyxoma

**DOI:** 10.1155/2022/5604460

**Published:** 2022-07-20

**Authors:** Daniel York, Smera Saikumar, Pavan Patel, Christian Edwards, Geossette Garcia, Hyder Naqvi

**Affiliations:** ^1^Colquitt Regional Medical Center, Sterling Center Women's Health, Moultrie GA 31768, USA; ^2^Philadelphia College of Osteopathic Medicine, South Georgia Campus, Moultrie, GA 31768, USA; ^3^Georgia South, Moultrie, GA 31788, USA

## Abstract

**Background:**

Aggressive angiomyxomas (AAs) are rare mesenchymal tumors that are histologically composed of myxoid stroma and vasculature. AAs are typically located in the pelvis and perineum and occur more frequently in females of reproductive age. *Case Presentation*. In this report, we outline a patient who had a paraurethral tumor with histopathology showing a circumscribed hypocellular lesion with myxoid stroma and abundant vasculature, consistent with the diagnosis of aggressive angiomyxoma. The mass was excised with resolution of symptoms and the patient was advised to continue close follow-up with her gynecologist and endocrinologist to monitor for recurrence.

**Conclusion:**

Due to its rarity, aggressive angiomyxomas are often misdiagnosed as cysts, hernias, lipomas, or cancerous lesions. Although benign, close follow-ups are crucial to monitor for recurrences or metastasis.

## 1. Introduction

Aggressive angiomyxoma (AA) is a mesenchymal tumor that is histologically evident by its appearance of myxoid stroma with abundant vasculature [1]. The characterization of “aggressive” denotes the tumor's high tendency to invade locally and its propensity to infiltrate the perivaginal and perirectal tissue [2]. Aggressive angiomyxoma is six times more prevalent in females of reproductive age than in males, with a peak incidence in the third to fifth decades of life [2]. In most of the reported cases, AA was observed in the pelvis or vulvar region. According to the MEDLINE query, it is a relatively rare tumor, with 309 occurrences reported between 1983 and 2022. Due to the rarity of aggressive angiomyxoma and the nonspecific symptoms that presents with AA, it is often misdiagnosed as a cyst, lipoma, hernia, or tumor, with the correct diagnoses made only after histological evaluation. Complete surgical excision with tumor-free margins is the first-line treatment. AA has a 30% recurrence rate with 71% of recurrences occurring within three years of resection [3]. Metastasis of AA is extremely rare with only 3 cases reported to date [4–6].

Although surgical excision of the tumor is the definitive treatment for AA, complications such as urinary incontinence and sexual dysfunction may occur due to the gynecological procedure [7]. Injury to the nearby anatomic structures can lead to weakened pelvic floor muscles which can cause stress incontinence. Studies have also shown that women who experience stress incontinence are less likely to participate in sexual intercourse and may even choose complete avoidance of sexual activity [8]. Due to these severe complications, appropriate care should be taken when removing the tumor. Frequent follow-ups are also crucial in patients with a history of AA to monitor for recurrences. In some instances, patients with a contraindication to surgical resection or those with tumor recurrences are given GnRH agonists to slow the growth of AAs [9].

## 2. Case Presentation

CG is a 31-year-old G1P1 premenopausal female with a past medical history significant for fibroids, who presented to the office for evaluation of a possible pelvic mass that had been present for eight months. She complained of vaginal itching and pelvic pressure with discomfort; however, she denied having vaginal discharge, pain, urinary urgency, urinary frequency, or vaginal odor. MRI performed on 11/15/21 reported a mass that was difficult to localize, possibly along the posterior vaginal wall.

**Figure 1:**
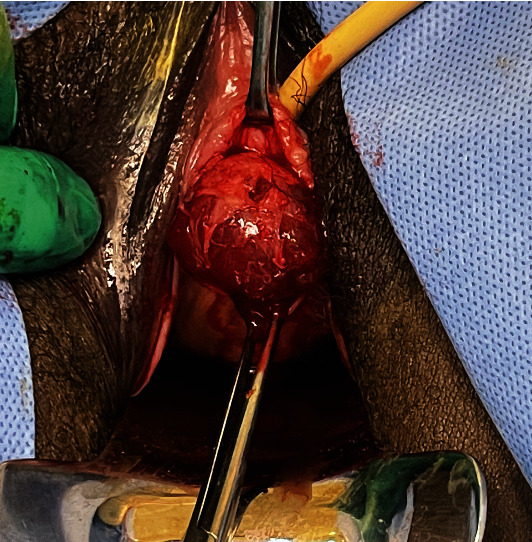
Gelatinous cystic mass protruding from the anterior vaginal wall.

**Figure 2:**
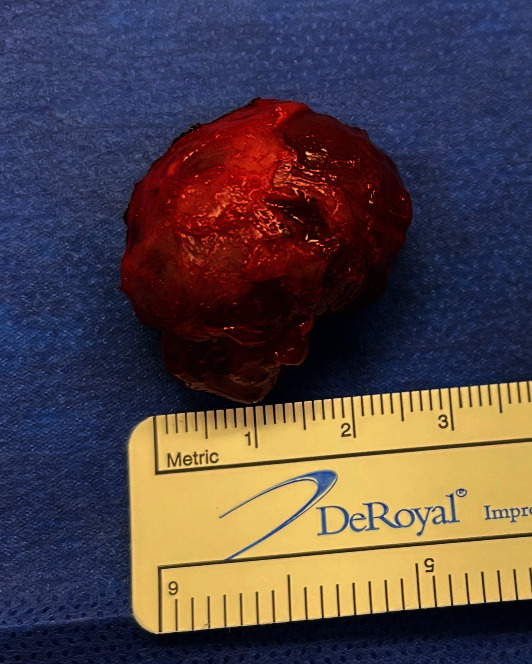
Gross specimen of the AA that was removed measuring about 2.75 cm in diameter.

**Figure 3:**
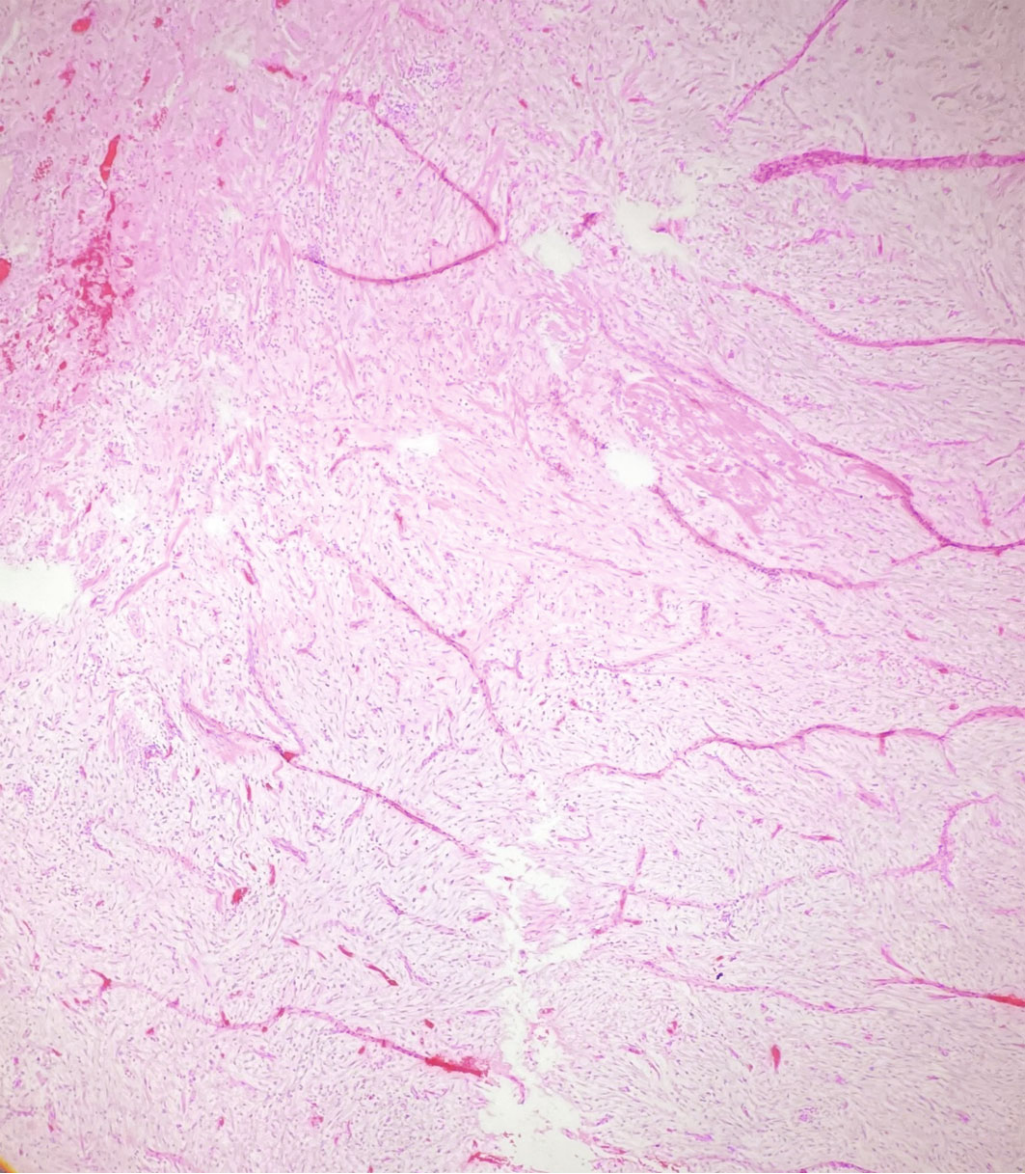
Aggressive angiomyxoma showing myxoid stroma and hypervascularity.

**Figure 4:**
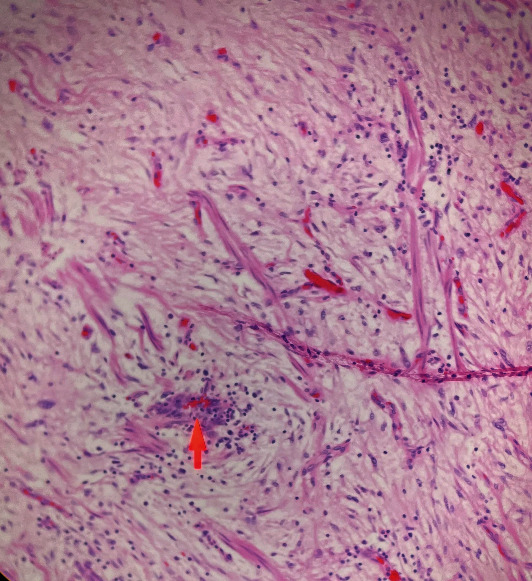
A hypocellular tumor comprising of myxoid stroma with areas of abundant vasculature.

Upon closer examination, the mass was noted to adhere to the anterior vaginal wall. All possibilities of urethral involvement were excluded before an attempt was made to drain the mass, which yielded 1 mL of thick gelatinous fluid. The fluid was sent for culture, which resulted in no bacterial growth. Excision in the operating room and marsupialization of the cyst was planned for a later date. Unfortunately, the patient was seen in the emergency department four days later due to worsening pain from the vaginal mass and dysuria. The patient also noted copious brown and purulent discharge with no foul odor over the past day. On physical examination, the patient had a swollen and tender spherical lesion adhered to the left anterior vaginal wall (Figure 1). She was taken to the operating room under general anesthesia for excision of the mass which measured 3.55 cm × 2.0 cm × 1.5 cm. The excision was successful and the mass was sent to pathology (Figure 2). The patient was then discharged with a Foley catheter in place, appropriate antibiotics, and pain medications. The pathology report described the mass as a circumscribed hypocellular lesion with myxoid stroma and abundant vasculature, with focally infiltrating fibroadipose tissue consistent with the diagnosis of aggressive angiomyxoma (Figures 3 and 4). The patient reported improvement in her symptoms at her follow-up appointment 4 days later, and the Foley catheter was removed at this time. She was advised to continue close follow-up with her gynecologist and an endocrinologist was incorporated for her care.

## 3. Discussion

Herein, we have discussed a case report of paraurethral aggressive angiomyxoma in a premenopausal woman. Angiomyxomas are typically slow-growing, asymptomatic vulvar masses that are sometimes unnoticed in patients for years [10]. There are two types of angiomyxoma: superficial and deep. Superficial angiomyxomas are more commonly found in men on the neck and trunk, with some cases reported on lower limbs, head, and genitalia as cutaneous tumors. In contrast, deep or aggressive angiomyxomas are six times more common in women and are typically found in the pelvis and perineum [1]. Macroscopically, AAs are described as edematous/gelatinous cystic masses containing mucoid content [9]. Microscopically, they are defined by the appearance of myxoid stroma with abundant vasculature [11].

AAs tend to be misdiagnosed as cysts, hernias, lipomas, or cancerous lesions due to their rarity and cystic mass-like appearance. AAs are most often benign; however, there have been 3 cases reported to date involving evidence of metastasis [4–6]. Surgery is the first-line treatment for AAs with the goal of complete resection [12]. When characterized as aggressive, angiomyxomas have a high recurrence rate of 30% due to their infiltrating qualities and absence of capsule, making it difficult for a complete resection with clear margins. 71% of recurrences happen within three years of the primary resection of AA. Although surgery is the preferred treatment, undergoing complex surgeries has been associated with urinary complications including stress incontinence. Symptoms such as surgery-induced incontinence sometimes do not develop until several years after the surgical procedure [7]. Studies have shown that urinary incontinence can also contribute to sexual dysfunction issues in women, including decreased frequency of intercourse and sometimes even a complete avoidance of sexual intercourse [8].

Despite possible complications, complete surgical excision with tumor-free margins is the preferred treatment. In cases where surgery is contraindicated, hormonal treatments may be used to slow the rate of growth of the AA [9]. Most AAs are either hormone-dependent or hormone-sensitive, leading to a higher likelihood of presentation in premenopausal women [9]. Hormonal therapies with continuous GnRH agonist or ER or PR antagonist can be beneficial to prevent reoccurrence. In primary tumors that are too large to resect, hormonal therapies have been shown to shrink the AAs to a manageable size where they can be resected with clear margins. Since the nature and the timing of the hormonal therapies remain unestablished, the treatment plan is best managed with a multidisciplinary team of OB/GYNs and endocrinologists to minimize the likelihood of recurrence [13].

## 4. Conclusion

Aggressive angiomyxoma is a rare neoplasm that is often misdiagnosed due to its cystic-like appearance. AAs should be considered as a differential diagnosis when a patient presents with a mass, particularly in the pelvis or perineum. Close observation is crucial to monitor for any recurrences or metastasis.

## Additional Points

*Teaching Point.* Aggressive angiomyxoma is an important differential to include in patients with a rapidly growing cystic mass that is confirmed by histopathological analysis. Treatment with a multidisciplinary team is essential for monitoring and preventing the recurrence of the tumor.

## References

[1] Gaurav A., Gill P., Khoiwal K., Chowdhuri S., Kapoor D., Chaturvedi J. (2020). Aggressive angiomyxoma of the vulva—a rare entity: case report and review of literature. *Journal of Reproduction, Contraception, Obstetrics and Gynecology*.

[2] Goyal L. D., Garg P., Badyal R., Bhalla S. (2022). Aggressive (deep) angiomyxoma of the vulva: a case report. *Journal of Medical Case Reports*.

[3] Chan Y. M., Hon E., Ngai S. W., Ng T. Y., Wong L. C. (2000). Aggressive angiomyxoma in females: is radical resection the only option. *Obstetricia Et Gynecologica Scandinavica*.

[4] Siassi R. M., Papadopoulos T., Matzel K. E. (1999). Metastasizing aggressive angiomyxoma. *The New England Journal of Medicine*.

[5] Blandamura S., Cruz J., Faure Vergara L., Machado Puerto I., Ninfo V. (2003). Aggressive angiomyxoma: a second case of metastasis with patient’s death. *Human Pathology*.

[6] Siddiqui S. F. (2020). Rare case of metastatic aggressive angiomyxoma-first case of renal metastasis. *Gynecology & Obstetrics Case report*.

[7] Ellström Engh M. A., Otterlind L., Stjerndahl J.-H., Löfgren M. (2006). Hysterectomy and incontinence: a study from the Swedish National Register for gynecological surgery. *Acta Obstetricia et Gynecologica Scandinavica*.

[8] Burzyński B., Kwiatkowska K., Sołtysiak-Gibała Z. (2021). Impact of stress urinary incontinence on female sexual activity. *European Review for Medical and Pharmacological Sciences*.

[9] Fetsch J. F., Laskin W. B., Lefkowitz M., Kindblom L. G., Meis-Kindblom J. M. (1996). Aggressive angiomyxoma: a clinicopathologic study of 29 female patients. *Cancer*.

[10] Linares Espinos E., Rengifo Abbad D., Van de Brule Rodriguez de Medina E., Osorio Cabello L., Areche Espiritusanto J., Carballido Rodriguez J. (2014). Aggressive pelvic angiomyxoma. *Archivos Espanoles De Urologia*.

[11] Sutton B. J., Laudadio J. (2012). Aggressive angiomyxoma. *Archives of Pathology & Laboratory Medicine*.

[12] Haldar K., Martinek I. E., Kehoe S. (2010). Aggressive angiomyxoma: a case series and literature review. *European Journal of Surgical Oncology (EJSO)*.

[13] Pannier D., Cordoba A., Ryckewaert T., Robin Y. M., Penel N. (2019). Hormonal therapies in uterine sarcomas, aggressive angiomyxoma, and desmoid-type fibromatosis. *Critical Reviews in Oncology/Hematology*.

